# Remittent Insanity Cured by Quinine

**Published:** 1856-02

**Authors:** H. W. Kreider


### ARTICLE IV.—*Remittent Insanity Cured by Quinine.* By Dr. H. W. Kreider.

Mrs. E. W. left home about the 15th of July, 1853, in company with her husband and some relations, with a view of visiting some friends in the Southern portion of the State, distant one hundred and twenty-five miles. After an absence of ten days they returned home, Mrs. W. complaining of little else than fatigue from her journey, the weather being extremely warm and roads very dirty, rendering the travelling by carrage and horses very unpleasant. But on three days after their return Mr. W. began to observe some actions in his wife that were unusual. They occurred only occasionally during the twenty-four hours, and he paid but little attention to it; but about the 1st of August he became alarmed on account of his wife, finding that she was restless during the night, slept but a little, up frequently, walking about for hours before daylight, when she would generally sleep, after which she appeared as well as usual until evening, when the symptoms of the preceeding night would again occur. She continued in this way until the morning of the 8th, when she was sized with violent insanity. She walked rapidly, but cautiously about the house, looking under beds, tables, &c.; saying that there were witches about the house, which she meant to find; also, picking up bits of paper, cloth, and articles of clothing, and burning them, in order, (as she said), to destroy the witches. She would also secrete knives and razors about her person. She also had a strong desire to escape from the house, to prevent which, the doors were closed and persons seated by them. Seeing resistance offered her escape, she made a leap through a small six light window, of eight by ten glass—a thing which would seem almost impossible—made her escape to the road in front of the house, and run half a mile before she could be overtaken; when brought back to the house, she was a furious maniac.

This morning, Saturday, August 8th, Dr. Gaddis was called, examined the patient, took 20 ℥ of blood from the arm, and returned home early Sunday morning. I was called in company with Dr. Gaddis to see the patient. She had spent a restless night, requiring four persons to watch and control her. She would not allow her husband to come into her presence, as she considered him the worst enemy she had. After obtaining from the husband of the patient a correct history of her health for some months past, we were unable to find any indications of disease, either of a physical or mental character, or any hereditary indications of this kind, but so far as known, the patient had enjoyed good health up to the attack. The patient's pulse this morning, 80 in the minute, tongue slightly coated, no thirst or heat of skin, or other indications of fever.

She complained of no pain whatever, and positively declared that there was nothing the matter with her. An emetic was ordered for the patient, to be followed by a cathartic, both of which operated freely. Some general directions were given as to the management of the patient, after which we returned home. Dr. G. declined further attendance on the part of the patient, as he had retired from practice.

Monday, August 7th, I was sent for this morning in great haste—the messenger stating that the patient had become so furious that they were scarcely able to restrain her from committing violence upon herself and others. I arrived at 10 o'clock, found the patient lying upon a bed, and held there by four men, making threats of the most violent kind; sometimes singing, and at other times hollowing, so as to be heard a great distance from the house.

Being unable to find any local disease or constitutional affections or any moral influence, sufficient to produce this state of the mind, I was at a loss to know what treatment to adopt. After some reflections upon the probable nature of the case, and carefully reviewing the patient's symptoms and actions from the commencement of the attack up to that time, and finding that there was a partial remission each morning of the insanity; the patient being more quiet and more easily controlled, although these symptoms lasted only two or three hours, and it being at a season of the year when diseases of a malanuous character were prevailing, I came to the conclusion that possibly this insanity might have the same cause for its origin—I come to the conclusion more particularly for the reason that the patient had been exposed in travelling to the excessive heat of the sun, which was very great at that time, (it being the dry summer of 1853), and the patient entirely unaccustomed to such exposure, consequently I came to the conclusion that it was a case of Remitting Insanity, depending for its origin upon the same influences that produced intermittent and remittent fevers. Treatment commenced Monday, August 7th, 12 o'clock, patient raving and furious, Chloroform was now administered to the patient until she was brought entirely under its influence, and quiet continued for half an hour, when it was allowed to pass off sufficiently to administer a powder composed of ji gr. sulp. morphine, jii grs. musk, and 5 grs. quinine. This prescription was repeated every three hours until three powders were taken, and the patient kept under the influence of chloroform near four hours, and until the quieting effects of the morphine began to be developed, which seemed to render the patient more manageable. A strait jacket was ordered, which was immediately made, and put upon the patient, so as to enable the attendant to control her; the musk, morphine and quinine were continued at intervals of four hours, until 40 grs. quinine were taken.

Tuesday, 3 o'clock, I had remained with the patient up to this time. No unpleasant symptoms were observed from the treatment or the large quantity of morphine she had taken; it producing only occasionally a few moments' sleep. The patient passed a better night than she had done the previous one, less violent in all her actions. This I attributed to the anodyne. I now prescribed powders containing i gr. sulp. morphine, and jii grs., to be given every four hours until my return next day.

Wednesday, 9 o'clock, evidently an improvement in the patient to day. She appears more calm, less inclined to try to injure her attendants; intervals of quiet during the night long. A saline cathartic was now given to the patient, which procured a moderate evacuation from the bowels. The quinine and morphine were again commenced at 9 o'clock, P. M., in the same doses as before, and same intervals.

Thursday, 2 o'clock, P. M., patient continues to improve, paroxysms of insanity less frequent and violent, particularly during the night. She could now easily be controlled; treatment continued until 6 o'clock the next morning, at which time it had been ordered to be discontinued during the day. The pulse during the whole time varied from 80 to 90 in the minute, seeming only to be increased by the intense excitement of mind, under which the patient labored. She eat nothing during the time, and only slept occasionally a few moments from the influence of morphine, being at times held upon her bed to restrain her, and at other times walked rapidly about her room.

Friday, 6 o'clock, P. M., patient has taken no medicine since

6 o'clock, A. M.—case reported by the attendant as follows: "Patient much better, rested well during the night, slept from 1 o'clock until 4 o'clock in the morning, eat a light breakfast, remained quiet, slept during greater part of the day, room kept quiet, and only one attendant required." After learning the history of the patient for the past twenty-four hours, I went to her room, enquired of her as to her health, she remarked that she was much better, immediately arose from her bed, seated herself in a large rocking chair, said she was well, and asked me very kindly, would I not allow her to be relieved of her strait jacket. It was immediately taken off, and the patient appearing perfectly sane, after a few moments' conversation, in which the patient exhibited no signs of mental derangement, she called for some nourishment, and after eating took her bed, feeling very much prostrated. No treatment recommended to-day, except rest and quiet.

Saturday, 6 o'clock, P. M., patient continued to improve, no unpleasant, mental symptoms during the last twenty-four hours, patient rested well during the night, and feels much refreshed to-day. The only treatment recommended for the patient in future was mild diet and rest. I visited the patient occasionally up to the 17th, she had so much improved that it was thought advisable that she should take exercise by riding in a carriage, and also visiting her friends, presuming it would hasten her recovery, by drawing her attention fron the late fearful scene through which she had passed.

Friday, 18th, Mr. Wyckoff and his wife left home for the town of Canton, with the view of visiting a brother living in that place, Mrs. W. appearing very much to enjoy the ride and visit, during the day. At tea time, they were conducted to a sleeping-room, immediately adjoining the public square of the town. The constant noise that was kept up in the street during the night, so annoyed Mrs. W. that she was unable to sleep, and became very much excited towards daylight in the morning. However, she appeared as well as usual during the day (19th.) The husband and wife occupied the same room the following night, the same noise and confusion occurring again, with the addition of the barking and howling of dogs. The want of sleep, and the intense excitement produced by the noise and confusion, brought on a recurrence of the insanity. In this condition, on the morning of the 20th, she was brought home.

I was immediately sent for, and found the patient as above stated. Her insanity was now not of a violent character. She could readily be controlled by her friends. Thirty grs. quinine, 5 grs. morphine, divided into five powders, and ordered to be given to the patient, commencing at 2 o'clock, P.M., one every three hours. Patient became quiet towards daylight; in the morning slept two hours; after which, she again appeared well and sane.

September 12th.—Mr. W. informed me to-day that his wife had been very restless for two or three nights, and was again deranged. Without visiting the patient, I prescribed morphine and quinine as above, in same proportions, and given in the same manner. The patient had been slightly deranged for two days after taking the medicine. The derangement immediately disappeared. The patient had two subsequent relapses, one on the 30th Sept., the other the 24th Oct., of mental derangement, which were promptly relieved by quinine and morphine.

